# The Hospitals' Handmaid

**Published:** 1924-01

**Authors:** 


### THE HOSPITALS' HANDMAID.

THE twenty-fourth annual meeting of Presidents of the League of Mercy was held at St. James's Palace, on Wednesday, December 19, the Grand President (the Prince of Wales) taking the chair. The League, which is an organisation by means of which persons able to give only small sums can subscribe to the Hospitals, makes impartial appeal to public benevolence and disinterested philanthropy for the support of all voluntary Hospitals within its area of operation, and distributes not less impartially to all Hospitals within its ambit. In the twenty-four years of its existence the League has contributed £339,000 to the London Hospitals, and much of this sum has been collected in small amounts, such as a penny a month, from the people of some of the poorest districts of London and other cities.

THE KING'S APPRECIATION.

At the commencement of his speech, the Prince of Wales read the following message from His Majesty the King:—"The Queen and I desire to convey to the Presidents, Vice-Presidents, members, and officers of the League of Mercy our warm appreciation of their sustained interest in this great and successful movement. More than a quarter of a century ago King Edward, himself a firm believer in the Voluntary System, instituted the Fund that bears his name and, two years later, the League of Mercy; and, though during this period many changes have taken place in the Hospital world, I feel that this system still commands the confidence and sympathy of all classes." The Prince then alluded to the payments made by patients to the Hospitals, a source of revenue which hardly existed when the League of Mercy was founded, a source which, though undoubtedly advantageous to the finances of individual Hospitals, could not be without influence on such a Central Collecting Agency, dependent on disinterested personal service, as the League of Mercy by its Charter was intended to be. Further, rival schemes such as ballots and similar competitions contrived to raise funds for charities have become popular, and in the case of some of them a considerable percentage of their contributions finds its way into the pocket of the professional organiser. No such charge can be levelled against the League of Mercy, since all contributions received by its members go without deduction to the maintenance of the Voluntary Hospitals. In conclusion the Prince, on behalf of all the members, paid a warm tribute to the gracious services rendered by the Lady Grand President, the Countess of Athlone, and presented her, with the special sanction of His Majesty, with the bar to the Order of the League.

THE YEAR'S TOTALS.

Sir Frederick Green, K.B.E., the Hon. Treasurer, stated that about £20,500 had been received in the past year, and though this amount fell slightly below that collected in 1921, it yet proved the enormous success which attended the efforts of members.

